# Usefulness of Preoperative Prognostic Nutritional Index in Secondary Spontaneous Pneumothorax

**DOI:** 10.1016/j.atssr.2024.06.016

**Published:** 2024-06-28

**Authors:** Toshio Shiotani, Kaoru Kondo, Shinichi Furukawa, Mototsugu Watanabe

**Affiliations:** 1 Department of Thoracic Surgery, NHO Iwakuni Clinical Center, Yamaguchi, Japan

## Abstract

**Background:**

The prognostic nutritional index has been identified as a predictor of postoperative outcomes in various fields. We investigated the usefulness of the prognostic nutritional index as a risk factor for postoperative complications in secondary spontaneous pneumothorax.

**Methods:**

In this retrospective study, patients who underwent surgery for secondary spontaneous pneumothorax were reviewed. The associations among the prognostic nutritional index, body mass index, and performance status were examined, and risk factors for postoperative complications were investigated in a multivariate analysis. In the subgroup analysis, on the basis of the receiver operating characteristics, we divided patients into high– and low–prognostic nutritional index groups and investigated the utility of the prognostic nutritional index for postoperative complications.

**Results:**

Of 91 patients who underwent surgery for secondary spontaneous pneumothorax, 34 patients with postoperative complications were included. The prognostic nutritional index was significantly positively and negatively correlated with body mass index (*P* < .001) and performance status (*P* < .001), respectively. Multivariate analysis revealed that a decreased prognostic nutritional index was an independent risk factor for postoperative complications (*P* = .0048). In the subgroup analysis, the postoperative outcomes, including the duration of chest tube placement (*P* = .042), hospital stay (*P* = .0014), complications (*P* = .0089), and mortality (*P* = .044), were significantly worse in the low– than in the high–prognostic nutritional index group.

**Conclusions:**

The prognostic nutritional index may be useful for evaluating the severity of secondary spontaneous pneumothorax in preoperative patients and may be a risk for postoperative complications.

## Visual Abstract

**Figure:**
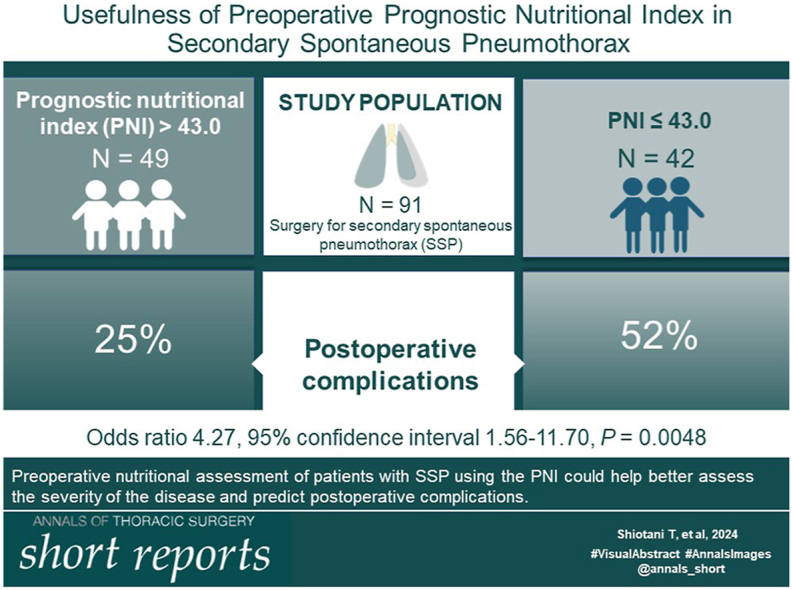

**In Short**

▪ The PNI could be a risk factor of postoperative complications in SSP.
▪ Early decision making to perform surgery in patients with SSP could preserve nutritional status and improve postoperative outcomes.

Secondary spontaneous pneumothorax (SSP) is defined as spontaneous pneumothorax in a patient with underlying lung disease or a significant smoking history of more than 50 years.^1^ Given the characteristics of SSP, the decision regarding surgery for SSP is sometimes difficult. However, few reports exist on postoperative outcome predictors in SSP.

The prognostic nutritional index (PNI), calculated using serum albumin and total lymphocyte count, ^2^ has recently gained attention as a useful index for assessing malnutrition status and predicting prognosis in several fields. However, there are still few reports in the field of thoracic surgery, including lung transplantation^2^ and lung cancer,^3^ and the relationship between the PNI and postoperative outcomes in SSP remains unclear. This study aimed to investigate the relationship between the PNI and postoperative outcomes in SSP.

## Patients and Methods

### Patients

The Institutional Review Board of the NHO Iwakuni Clinical Center (Yamaguchi, Japan) approved the study protocol (No. 0360; February 1, 2024) and waived the requirement for written informed consent. All methods were performed in accordance with relevant guidelines and regulations. This retrospective cohort study involved patients who underwent surgery for SSP at the NHO Iwakuni Clinical Center between January 2010 and December 2023. SSP was diagnosed on the basis of previous reports.^1^ Preoperative and intraoperative characteristics and postoperative outcomes were assessed.

### Data Collection

Serum albumin levels and total lymphocyte counts were obtained within 3 days of surgery. The PNI was calculated using the following equation:

$$PNI=10\times albumin\;\left(g∕dL\right)+\left(0.005\times total\;lymphocyte\;count\;\left[∕{mm}^{3}\right]\right)$$

The psoas muscle mass index (PMI), which is associated with sarcopenia, was assessed using a previously described method.^4^

### Statistical Analysis

All statistical analyses were conducted using EZR, a graphic user interface for R software (R Foundation). The Spearman rank correlation coefficient was used for association analysis. Receiver operating characteristic (ROC) analyses using the Youden index were performed to determine the cutoff value of the discriminative abilities of preoperative continuous variables for postoperative complications. On the basis of the ROC analysis, the patients were divided into high- and low-PNI groups. The Mann-Whitney *U* test for continuous variables and the Pearson χ^2^ test for categoric variables were used to analyze the patients’ characteristics between the groups. Risk factors for the development of grade 2 or higher postoperative complications, according to the Clavien-Dindo classification,^5^ were analyzed by multivariate analysis using logistic regression analysis with the stepwise *P* value method. Missing data have not been replaced. *P* values <.05 indicated statistical significance.

## Results

Of the 91 patients who underwent surgery for SSP, 34 experienced postoperative complications (Table 1). The cutoff values were defined as follows: age, 64 years (area under the curve [AUC], 0.50); body mass index (BMI), 18.5 kg/m^2^ (AUC, 0.53); performance status (PS), 2 (AUC, 0.55); PNI, 43.0 (AUC, 0.67); PMI, 4.35 (AUC, 0.55); and time since onset to operation, 8 days (AUC, 0.59) by ROC curve analysis. The odds of postoperative complications were significantly higher in patients with a PNI ≤43.0 (*P* = .0089) or interstitial lung disease comorbidity (*P* = .013) on the basis of univariate analysis. Multivariate analysis revealed that the PNI was an independent risk factor for postoperative complications (*P* = .0048) (Table 2).

**Table 1:** Patient Characteristics

Characteristics	Values (N = 91)
Preoperative variables	
Age, y	75 (47-93)
Sex, male	81 (89)
Body mass index, kg/m^2^	19.8 (14.1-27.8)
Performance status	1 (0-4)
Smoking ≥20 pack-years	70 (77)
Home oxygen therapy	7 (7.7)
Use of glucocorticoid	7 (7.7)
Location of pneumothorax, left	47 (52)
Prognostic nutritional index	43.3 (21.3-67.1)
Psoas muscle mass index, cm^2^/m^2^	3.84 (1.25-8.20)
Comorbidities	
Cardiovascular disease	21 (23)
Cerebroneurovascular disease	5 (5.5)
Cancer	20 (22)
Diabetes mellitus	8 (8.8)
Chronic obstructive pulmonary disease	26 (29)
Interstitial lung disease	10 (11)
Others	26 (22)
Time since onset to operation, d	4 (0-72)
Preoperative chemical pleurodesis	13 (14)
Intraoperative variables	
Approach	
Video-assisted thoracic surgery	67 (74)
Thoracotomy	24 (26)
Operative time, min	109 (40-305)
Procedure for air leak points	
Partial resection and covering	59 (65)
Suturing or ligation, and covering	25 (27)
Cautery and covering	4 (4.5)
Covering only	3 (3.5)
Postoperative variables	
Duration of chest tube placement, d	2 (1-23)
Hospital stay, d	7 (3-95)
Complications	34 (41)
Prolonged air leak	16 (18)
Respiratory failure	9 (9.9)
Empyema	2 (2.2)
Pneumonia	4 (4.4)
Others	9 (9.9)
Mortality during hospital stay	4 (4.4)
Recurrence of pneumothorax	9 (9.9)

Data are presented as n (%) or median (range).

**Table 2:** Univariate and Multivariate Analyses of the associations Between Preoperative Variables and Postoperative Complications

Variables	Univariate Analyses		Multivariate Analysis	
	Odds Ratio (95% CI)	*P* Value	Odds Ratio (95% CI)	*P* Value
Age, y				
≥64	1.87 (0.31-20.05)	.71	...	...
Sex				
Male	0.88 (0.19-4.61)	1.00	...	...
Body mass index, kg/m^2^				
≤18.5	1.84 (0.70-4.91)	.18	...	...
Performance status				
≥2	1.27 (0.39-3.97)	.70	...	...
Smoking ≥20 pack-years	0.80 (0.24-2.79)	.78	...	...
Home oxygen therapy	2.38 (0.37-17.32)	.42	...	...
Use of glucocorticoid	2.38 (0.37-17.32)	.42	...	...
Prognostic nutritional index				
≤43.0	3.34 (1.2–9.13)	.0089	4.27 (1.56-11.70)	.0048
Psoas muscle mass index, cm^2^/m^2^				
≤4.35	1.83 (0.63-5.69)	.24	...	...
Comorbidity				
Interstitial lung disease	4.58 (0.95-29.63)	.036	...	...
Time since onset to operation, d				
≥8	2.54 (0.86-7.69)	.077		...
Preoperative chemical pleurodesis	1.06 (0.25-4.07)	1.00	...	...

Patients were divided into 2 groups on the basis of a PNI cutoff value of 43.0 (PNI > 43.0 [high-PNI group, n = 49]; PNI ≤ 43.0 [low-PNI group, n = 42]) (Table 3). Among the preoperative variables, compared with the high-PNI group, the low-PNI group had significantly lower BMI (*P* < .001), PS (*P* = .0013), and PMI (*P* = .0099), as well as a longer time since onset to surgery (*P* = .028). Concerning the procedure for air leak points, the high-PNI group had significantly more partial resections (*P* = .0020) and less suturing or ligation (*P* = .018) than the low-PNI group. The low-PNI group had longer postoperative tube placement (*P* = .042), a longer postoperative hospital stay (*P* = .0014), and more postoperative complications (*P* = .0089) than the high-PNI group. All patients who died during their hospital stay after surgery for SSP were from the low-PNI group (*P* = .044).

**Table 3:** Perioperative Variables of Secondary Spontaneous Pneumothorax According to the Preoperative Prognostic Nutritional Index

Variables	High PNI (n = 49)	Low PNI (n = 42)	*P* Value
Preoperative variables			
Age, y	75 (47-92)	77 (64-93)	.061
Sex, male	46 (94)	35 (83)	.18
Body mass index, kg/m^2^	20.8 (14.8-27.8)	18.3 (14.1-25.7)	<.001
Performance status	0 (0-4)	1 (0-4)	.0013
Smoking ≥20 pack-years	43 (88)	27 (64)	.061
Home oxygen therapy	3 (6.1)	4 (9.5)	.70
Use of glucocorticoid	3 (6.1)	4 (9.5)	.70
Location of pneumothorax, right	29 (59)	18 (43)	.14
Psoas muscle mass index, cm^2^/m^2^	4.10 (1.43-8.20)	3.54 (1.25-7.95)	.0099
Comorbidities			
Cardiovascular disease	12 (25)	9 (21)	.81
Cerebroneurovascular disease	3 (6.1)	2 (4.8)	1.00
Cancer	10 (20)	10 (24)	.80
Diabetes mellitus	5 (10)	3 (7.1)	.72
Chronic obstructive pulmonary disease	13 (27)	13 (31)	.65
Interstitial lung disease	3 (6.1)	7 (17)	.18
Others	17 (35)	9 (21)	.24
Time since onset to operation, d	3 (0-72)	5 (1-25)	.028
Preoperative chemical pleurodesis	6 (12)	7 (17)	.56
Intraoperative variables			
Approach			
Video-assisted thoracic surgery	39 (80)	28 (67)	.23
Thoracotomy	10 (20)	14 (33)	
Operative time, min	105 (40-295)	115 (40-305)	.15
Procedure for air leak points			
Partial resection and covering	39 (80)	20 (48)	.0020
Suturing or ligation, and covering	8 (16)	17 (41)	.018
Cautery and covering	1 (2.0)	3 (7.2)	.33
Covering only	1 (2.0)	2 (4.8)	.59
Postoperative variables			
Duration of chest tube placement, d	1 (1-23)	2 (1-15)	.042
Hospital stay, d	6 (3-26)	10 (3-95)	.0014
Complications	12 (25)	22 (52)	.0089
Prolonged air leak	5 (10)	11 (26)	.057
Respiratory failure	1 (2.0)	8 (19)	.011
Empyema	0 (0)	2 (4.8)	.21
Pneumonia	1 (2.0)	3 (7.1)	.33
Others	5 (10)	4 (9.5)	1.00
Mortality during hospital stay	0 (0)	4 (9.5)	.044
Recurrence of pneumothorax	4 (8.2)	5 (12)	.73

Data are presented as n (%) or median (range).

PNI, prognostic nutrition index.

## Comment

This study identified a low preoperative PNI (<43.0) as a risk factor for postoperative complications in SSP. Moreover, compared with the low-PNI group, the high-PNI group had a significantly higher BMI and better PS. On the basis of these findings, the PNI could indicate the severity of a patient’s preoperative condition and nutritional status, thus predicting postoperative complications in SSP.

Malnutrition negatively affects immune function, inflammation, and wound healing. Moreover, in patients with lung diseases, malnutrition results in quantitative and functional changes in skeletal and respiratory muscles, thereby further compromising their condition and affecting quality of life and survival.^6^ In this study, although the long-term outcomes were unknown, there were significant differences between the high- and low-PNI groups concerning the PMI, postoperative complications (especially respiratory failure), and postoperative mortality during the hospital stay. Further studies on the relationship between malnutrition and postoperative outcomes in SSP are warranted.

The PNI also affected the control of air leaks during surgery. This relationship could be explained by the association between nutritional status and the degree of emphysematous change. Patients with severe or very severe chronic obstructive pulmonary disease (COPD) often have diffuse emphysematous changes, and approximately one-fourth of these patients are malnourished.^6^ Unfortunately, the severity of COPD was not assessed in this study. However, given previous findings,^6^ the severity of COPD could have been higher in the low-PNI group than in the high-PNI group. Stapling lung tissue with severe emphysematous changes can cause adverse events,^7^ and this could explain why the low-PNI group had a lower proportion of partial resection of air leak points compared with the high-PNI group.

The PNI may be affected by preoperative patient characteristics other than nutritional status. A prolonged preoperative drainage period can decrease serum total protein and increase the postoperative hospital stay.^7^ Decreased serum total protein levels could result in a lower PNI, and the longer preoperative chest tube placement in the low PNI group may have affected the postoperative outcomes in this study. Given the current results, early decision making to perform surgery could preserve a higher PNI and lead to better postoperative outcomes.

This study has several limitations. First, it was a retrospective study conducted at a single institution with relatively few patients. Second, although several indicators of nutritional status have been reported, this study did not examine indicators other than the PNI. Third, although all participants in our study were Japanese, the physical and nutritional characteristics of the patients differed among the countries. However, our findings provide valuable information that PNI may be a risk factor for postoperative complications in SSP.

In conclusion, the PNI of patients with SSP could be an independent prognostic factor for postoperative complications. Preoperative nutritional assessment of patients with SSP by using the PNI could aid in the evaluation of disease severity and predict postoperative complications.
